# Wnt5a deficiency in osteocalcin-expressing cells could not alleviate the osteoarthritic phenotype in a mouse model of post-traumatic osteoarthritis

**DOI:** 10.22038/IJBMS.2024.71417.15527

**Published:** 2024

**Authors:** Lin-Jie Feng, Xin-Hao Fan, Li-Tao Shao, Yun-Peng Zhang, Yun-Peng Hu, Yue Li, Xiao-Li Hou, Liu Zhang, Fa-Ming Tian

**Affiliations:** 1 Department of Orthopedic Surgery, Hebei Medical University, Shijiazhuang, Hebei, P.R. China; 2 School of Public Health, North China University of Science and Technology, Tangshan, Hebei, P.R. China; 3 Trauma Department of the 982 Hospital of The Joint Service Support Force of the Chinese people’s Liberation Army, Tangshan, Hebei, P.R. China; 4 Department of Stomatology, Kailuan General Hospital, Tangshan, China; 5 Department of Orthopedic Surgery, Emergency General Hospital, Beijing, 100028, China

**Keywords:** Cartilage, Osteoarthritis, Osteoblast, Subchondral bone, Wnt5a

## Abstract

**Objective(s):**

Wnt5a, which regulates the activities of osteoblasts and osteoclasts, is reportedly overexpressed in osteoarthritis (OA) tissues. The purpose of this study was to elucidate its role in the development of OA by deleting Wnt5a in osteocalcin (OCN)-expressing cells.

**Materials and Methods:**

Knee OA was induced by anterior cruciate ligament transection (ACLT) in *OCN-Cre;Wnt5a*^fl/fl^ knockout (Wnt5a-cKO) mice and control littermates. Eight weeks after surgery, histological changes, cell apoptosis, and matrix metabolism of cartilage were evaluated by toluidine blue, TUNEL staining, and im-immunohistochemistry analyses, respectively. In addition, the subchondral bone microarchitecture of mice was examined by micro-computed tomography (micro-CT).

**Results:**

Histological scores show substantial cartilage degeneration occurred in ACLT knees, coupled with decreased collagen type II expression and enhanced matrix metalloproteinase 13 expression, as well as higher proportions of apoptotic cells. Micro-CT results show that ACLT resulted in decreased bone mineral density, bone volume/trabecular volume, trabecular number, and structure model index of subchondral bones in both Wnt5a-cKO and control littermates; although Wnt5a-cKO mice display lower BMD and BV/TV values, no significant difference was observed between Wnt5a-cKO and control mice for any of these values.

**Conclusion:**

Our findings indicate that Wnt5a deficiency in OCN-expressing cells could not prevent an osteoarthritic phenotype in a mouse model of post-traumatic OA.

## Introduction

Osteoarthritis (OA), the most common form of chronic degenerative joint disorder and a major cause of disability in older individuals, is characterized by articular cartilage degeneration, inflammation of the synovial membrane, and abnormal bone remodeling with osteophyte formation. Increasing evidence indicates that abnormal subchondral bone remodeling strongly contributes to the development of OA (1, 2). During the pathogenesis of OA, subchondral bone sclerosis is reportedly associated with and potentially causes cartilage degeneration (3, 4).

Previous studies showed that some bone metabolism-modulating agents or strategies retard OA development by limiting pathologic changes involving subchondral bone remodeling (5-8). As such, the crosstalk and critical interlinked signaling pathways between cartilage and bone have become key targets for the development of effective strategies to prevent and treat OA (9). Emerging evidence indicates the involvement of noncanonical Wnt signaling pathways in the development of OA (10); these pathways are predominantly activated by Wnt5a and classified into Wnt/Ca^2+^ and planar cell polarity pathways. Briefly, these pathways are mediated through either the induction of phospholipase C/protein kinase C/Ca^2+^ or calmodulin-sensitive protein kinase II pathways to activate transcription factor nuclear factor associated with T cells (NFAT) or disheveled, which triggers Rho/Rho-associated kinase and Rac/c-Jun N-terminal kinase (JNK) signaling to regulate downstream effectors. These complex signaling events have been implicated in various inflammatory diseases, including OA (11, 12).

Wnt5a expression in articular cartilage has been positively correlated with progressive damage of the knee joints of patients with OA (10). Moreover, subchondral osteoblasts harvested from the tibial plateaus of patients with OA displayed a fivefold increase in Wnt5a expression, as well as increased alkaline phosphatase (ALP) activity and osteocalcin (OCN) levels, compared with normal osteoblasts. Inhibiting Wnt5a expression partially prevented this abnormal mineralization, OC secretion, and ALP activity in OA osteoblasts (13). Increases in *Cxcl12* and *Rankl* gene levels induced by JNK and Ca^2+^/NFAT signaling pathways lead to activation of osteoclast differentiation and enhanced subchondral bone turnover (14). We, therefore, hypothesized that the Wnt5a signaling pathway mediates a pathologic interaction between osteoblasts and chondrocytes following trauma to the joint, triggering both pathologic subchondral bone remodeling and cartilage catabolic metabolism. 

The Cre-loxP system has been widely used to investigate gene function in a tissue- or cell-specific manner. Expressed mainly in mature osteoblasts, as well as hypertrophic chondrocytes in osteoarthritic cartilage (15), OCN was used as a Cre driver. The present study aimed to elucidate the role of Wnt5a in OCN-expressing cells during the development of OA in terms of changes in cartilage and subchondral bone by establishing an anterior cruciate ligament transection (ACLT) model in *OCN-Cre;Wnt5a*^fl/fl^ transgenic mice.

## Materials and Methods

***Generation of mice with osteoblast-specific Wnt5a knockout***

To delete *Wnt5a* in OCN-expressing cells, female mice homozygous for the *Wnt5a* gene flanked by loxP sites (The Jackson Laboratory, Stock No: 026626) were bred with male transgenic mice expressing Cre recombinase under the control of the OCN promoter (OCN-Cre) (The Jackson Laboratory, Stock No: 019509). *Wnt5a*^fl/-^*;OCN-Cre* mice were obtained in the first generation. Offspring of the second generation with a *Wnt5a*^fl/fl^*;OCN-Cre* genotype were defined as conditional knockout (cKO) mice, while littermates that did not express Cre recombinase but were homozygous for Wnt5a flanked by loxP served as controls (Ctrl).

***ACLT surgery to establish the OA model***

To avoid sex-related differences in disease severity, we used only male mice in this experiment. When osteoblast-specific Wnt5a knockout (cKO) mice and their littermate control mice (Ctrl)(18-23 g) reached 8 weeks of age, ACLT (Ctrl-ACLT/cKO-ACLT) surgery was performed on the right knees by a single surgeon. The contralateral knee joints underwent a sham operation using the same approach without any ligament transection (Ctrl-sham/cKO-sham). All mice received water and complete pelleted food *ad libitum* and were caged in groups (n=3-6 mice per cage) with unrestricted movement until sacrificing 8 weeks after surgery. All procedures were performed according to the protocol approved by the Animal Care and Use Committee.

***Histological assessments***

The bilateral knee joints were harvested from Ctrl and cKO mice, cleaned to remove the soft tissues, fixed in 10% formalin solution, decalcified with 10% EDTA for 8 weeks, dehydrated in a graded series of ethanol solutions, and embedded in paraffin wax according to standard protocols. The tissue blocks were then sectioned in the coronal plane to 6 μm in thickness and stained with toluidine blue for histological observation. In detail, we initially counted 80 10-μm sections and 20 6-μm sections after the first cutting of the tibia plateau and then collected sections for histological and immunohistochemistry. Degenerative changes and thickness of the articular cartilage of the medial part of the tibia plateau were evaluated in two sections of each sample by two or three blinded observers. The modified Osteoarthritis Research Society International (OARSI) semiquantitative scoring system for osteoarthritic damage based on the work of Chambers and Carlson (Table 1)(16, 17) was used to evaluate tissue. 

***Immunohistochemistry***

Immunohistochemical localization of Wnt5a (1:200; Abcam, Inc., Cambridge, UK), collagen type II (Col2a1) (1:300; DSHB Hybridoma Product II-II6B3, from Linsenmayer TF), aggrecan (1:300; Abbiotec LLC, San Diego, CA, USA), ADAMTS-4 (1:500; Boster Co., Ltd., Wuhan, China), and matrix metalloproteinase (MMP)-13 (1:1000; Boster Co., Ltd., Wuhan, China) were performed for paraffin sections. Briefly, deparaffinized tissue sections were rehydrated with ethanol, digested with 0.05% trypsin, treated with 3% hydrogen peroxide, and incubated with primary antibodies overnight at 4 ^°^C. IgG isotype controls were included to verify antibody specificity. Next, DAB staining was conducted in accordance with protocols provided with the PV-6000 DAB Detection Kit and ZLI-9018 DAB Kit (all from ZSGB-BIO Corp., China). Finally, sections were counterstained with hematoxylin. Immunohistochemical images of the medial part of the tibia plateau from two sections of each sample (including cartilage and partial sub-chondral bone) were captured with a BX53 microscope (Olympus, Tokyo, Japan) and then semiquantitatively analyzed with Image-Pro Plus version 6.0 software (Media Cybernetics, Rockville, MD, USA). Immunohistochemistry results are shown as the average intensity of the optical density (IOD/mm^2^), according to a previously described method (18).

***TUNEL staining***

Terminal deoxynucleotidyl transferase dUTP nick end labeling (TUNEL) staining was used to detect apoptotic chondrocytes in cartilage with an ApopTag® Peroxidase *In Situ* Apoptosis Detection Kit (Merck Millipore, Burlington, MA, USA) according to the manufacturer’s instructions. The processing flow of paraffin sections was the same as employed for immunohistochemistry. Finally, TUNEL staining images were captured with a BX53 microscope and TUNEL-positive cells were evaluated as the percentage of cartilage cells determined by Image-Pro Plus software.

***Micro-computed tomography (micro-CT) analysis***

Changes in subchondral bone architecture of the knee joints were scanned by high-resolution micro-CT (SkyScan1176, Bruker, Belgium) with a resolution of 9.5 μm per voxel. The trabecular bone of the tibia subchondral bone at the cross-sectional level, excluding the cortical shell, was manually selected as the region of interest at the tibial plateau. Bone mineral density (BMD), bone volume/trabecular volume (BV/TV), trabecular thickness (Tb.Th), trabecular number (Tb.N), trabecular separation (Tb.Sp), and the structure model index (SMI) were calculated. For subchondral bone plate thickness (SBP.Th) analysis, cortical bone of the tibia plateau on the central loading regions of the medial part was calculated, with an average of seven measurements of three slices made for each sample. 

***Statistical analysis***

All analyses were performed using SPSS software (SPSS 22.0, SPSS, Inc.; Chicago, IL, USA). G*Power 3.1.9.2 software (http://www.gpower.hhu.de/) was used for sample size calculation before the study was conducted, and the results indicated that a sample size of six mice per group would be well-powered to detect changes in our study. Specifically, in an *a priori* power analyses based on BMD, input parameters specifying a two-tailed t-test, the large effect size of Cohen’s d (commonly just referred to as d)=1.8, α=0.05, pre-specified power (1-β)=0.8, and an allocation ratio of N2/N1=1 resulted in a sample size of N=6 for each group. Thus, we chose six mice per group. The above-mentioned d=1.8 was defined according to the results of our preliminary experiments. The Shapiro-Wilk test was used to assess whether the results presented a normal distribution, and the means were compared by one-way analysis of variance (ANOVA). Kruskal-Wallis H and Dunn-Bonferroni *post hoc* tests were used for comparisons of the histological scores of each group. The results are shown as the mean±standard error of the mean (SEM), with the alpha level set at 0.05.

## Results

***Histological analysis of cartilage tissues***

**Figure 1:**
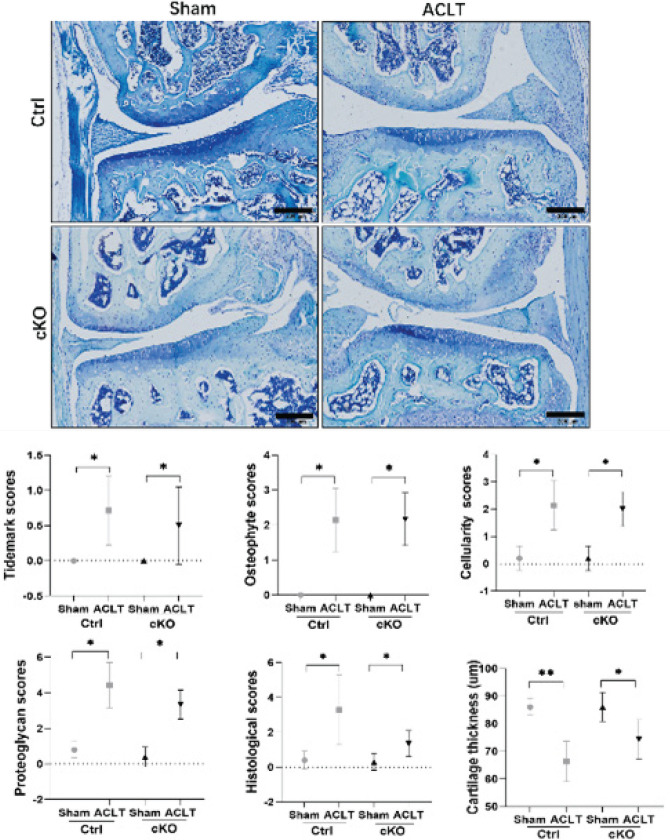
Toluidine blue staining, histological scores, and cartilage thicknesses in sham and ACLT knees of Ctrl and cKO mice

In the contralateral knees of Ctrl and cKO mice, the articular cartilage of the tibial plateau was nearly intact, the structures of chondrocytes were normal, and the extra-cellular matrix stained by toluidine blue was evenly distributed. Distinct proliferation of the articular soft tissue and cartilage degeneration were observed in Ctrl-ACLT and cKO-ACLT knees compared with their contralateral knees. Proteoglycan, tidemark, cellularity, osteophyte, and histological scores were higher in Ctrl-ACLT and cKO-ACLT knees compared with their contralateral knees (all *P*<0.05). The cartilage thickness of Ctrl-ACLT and cKO-ACLT knees was significantly decreased compared with their contralateral knees (*P*<0.01; *P*<0.05). However, there was no significant difference between ACLT or contralateral knees in Ctrl and cKO mice (*P*>0.05)(Figure 1).

***Immunohistochemical assessments***

**Figure 2:**
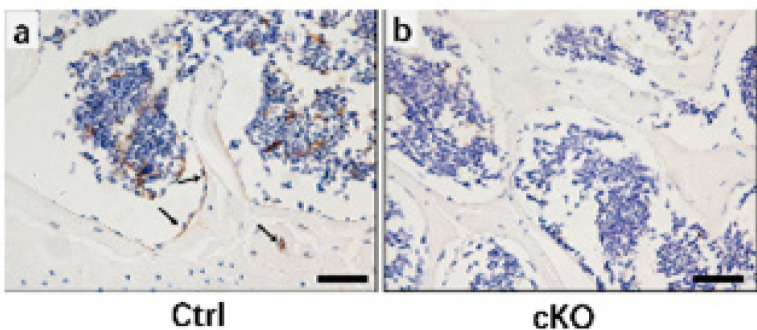
Wnt5a expression in Ctrl and cKO mice

Wnt5a-positive cells were found in the bone osteoblasts of sham mouse sections, but Wnt5a expression was not observed in the osteoblasts of cKO mouse sections (Figure 2). 

**Figure 3:**
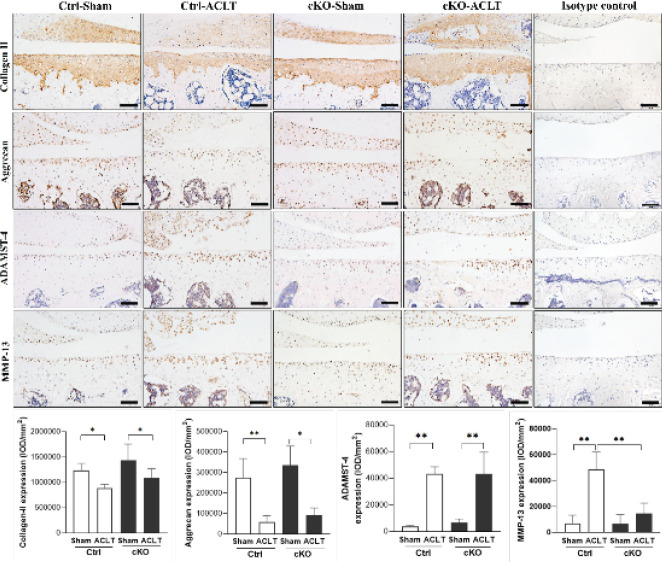
Immunohistochemical assays for collagen type II, aggrecan, ADAMTS-4, and MMP-13 in the cartilage of Ctrl and cKO mice

**Figure 4:**
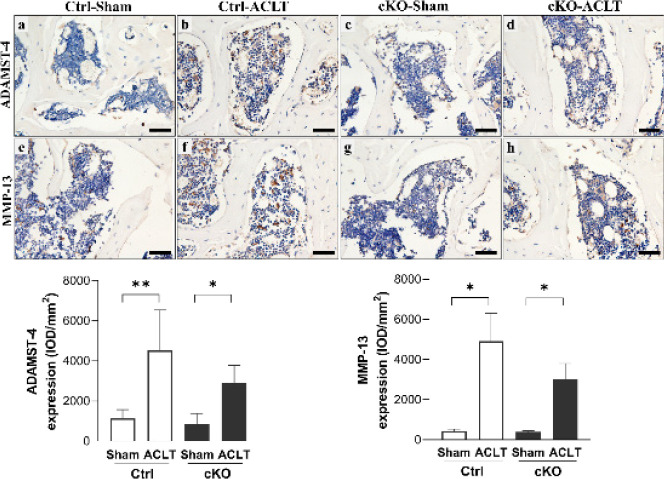
Immunohistochemical assays for ADAMTS-4 and MMP-13 in the subchondral bone of Ctrl and cKO mice

Col2a1 and aggrecan expression levels were significantly lower in ACLT knees compared with their contralateral knees in both Ctrl and cKO mice (*P*<0.05; *P*<0.01). Furthermore, no significant changes in Col2a1 or aggrecan expression were observed between Ctrl and cKO mice, either in ACLT or contralateral knees (*P*>0.05). Significantly higher MMP-13 expression levels were observed in the cartilage of Ctrl-ACLT knees compared with their contralateral knees (*P*<0.01). The expression level of MMP-13 in subchondral bone and expression levels of ADAMTS-4 in the cartilage and subchondral bone were significantly higher in Ctrl-ACLT and cKO-ACLT knees compared with their contralateral knees (*P*<0.05, *P*<0.05; *P*<0.01, *P*<0.01; *P*<0.01, *P*<0.05, respectively)(Figures 3 and 4). 

***TUNEL staining assessment***

**Figure 5:**
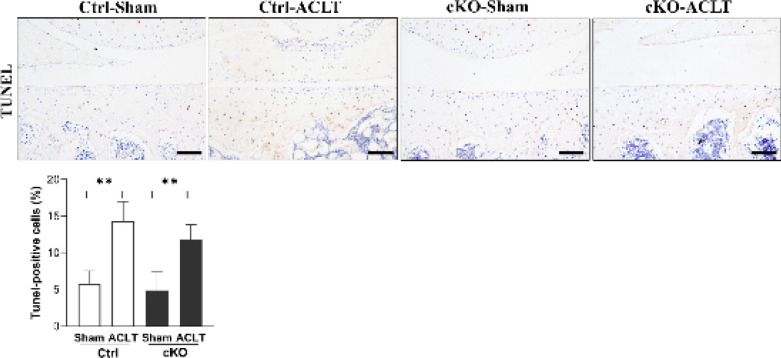
TUNEL staining analysis of Ctrl and cKO mice

Percentages of TUNEL-positive cells in Ctrl-ACLT and cKO-ACLT knees were higher than in their contralateral sham knees (all *P*<0.01), and there were no significant changes in levels of TUNEL-positive cells in cKO-ACLT knees compared with Ctrl-ACLT knees (*P*>0.05)(Figure 5).

***Micro-CT parameters of subchondral bone***

**Figure 6:**
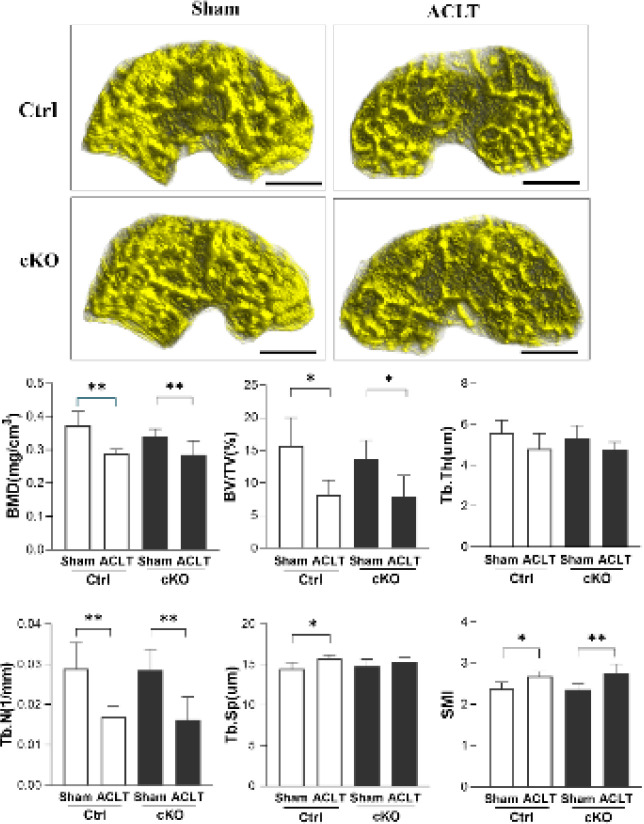
Micro-CT images of the subchondral bone of sham and ACLT knees in Ctrl and cKO mice

**Figure 7:**
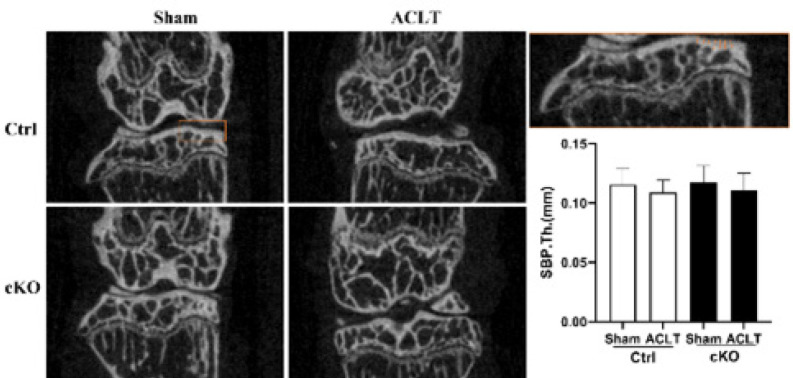
Subchondral bone plate thickness of the medial part of the tibia plateau in sham and ACLT knees in Ctrl and cKO mice

The results show significant differences in the BMD, BV/TV, Tb.N, Tb.Sp, and SMI values of Ctrl-ACLT knees compared with their contralateral knees (*P*<0.01, *P*<0.05, *P*<0.01, *P*<0.05, and *P*<0.05, respectively). The results also show that no significant changes in BMD, BV/TV, Tb.N, Tb.Sp, SMI, or Tb.Th values occurred between Ctrl-ACLT and cKO-ACLT knees (all *P*>0.05). For cKO mice, there were significant differences in BMD, BV/TV, Tb.N, and SMI values, but not Tb.Sp, between ACLT knees and their contralateral knees (*P*<0.01, *P*<0.05, *P*<0.01, and *P*<0.01, respectively)(Figure 6). No significant difference in SBP.Th was observed between ACLT knees and their contralateral knees in cKO mice and their control littermates (*P*<0.05) (Figure 7).

**Table 1:** The modified Osteoarthritis Research Society International (OARSI) semi-quantitative scoring system for osteoarthritic damage

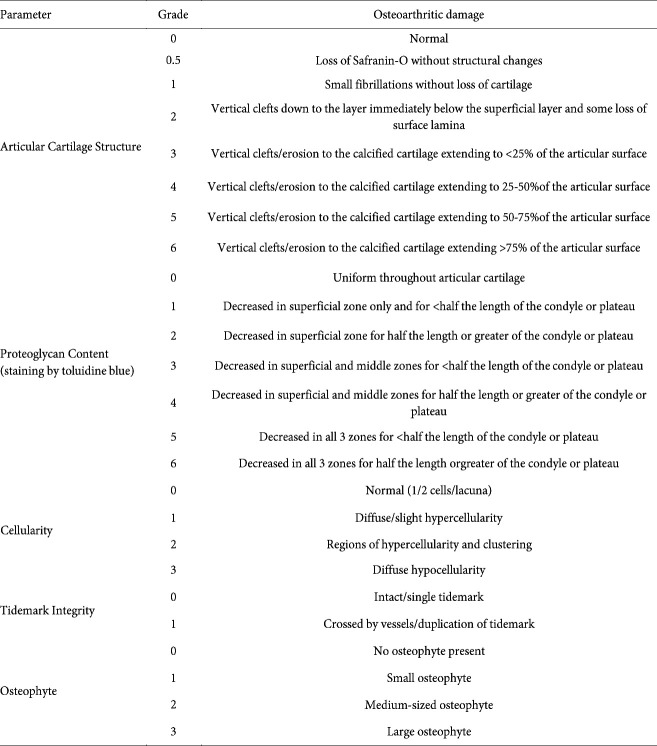

Measurements of histological score and cartilage thickness. Bar=200 μm.*P<0.05; **P<0.01

ACLT: Anterior cruciate ligament transection; Ctrl: control; cKO: conditional knockout

(a) Ctrl mice, (b) cKO mice. Arrows indicate Wnt5a-positive cells. Bars = 50 μm

Ctrl: control; cKO: conditional knockout

Data are represented as the mean±SEM. Bars=100 μm. *P<0.05; **P<0.01

Ctrl: control; cKO: conditional knockout

Data are represented as the mean±SEM. Bars=100 μm. *P<0.05; **P<0.01

Ctrl: control; cKO: conditional knockout

Data are represented as the mean±SEM. Bars=100 μm. *P<0.05; **P<0.01

Ctrl: control; cKO: conditional knockout

Micro-architecture parameters are represented as the mean±SEM. *P<0.05; **P<0.01

ACLT: Anterior cruciate ligament transection; Ctrl: control; cKO: conditional knockout

Data are represented as the mean±SEM

## Discussion

In the present study, osteoarthritic degeneration in the knee joint in mice was confirmed at 8 weeks after ACLT (a well-accepted method to establish an OA model that mimics unstable or post-traumatic joint degeneration (19-21)) via histological analysis, OARSI score in terms of extracellular matrix content, cartilage thickness, chondrocyte apoptosis, and osteophyte formation. Importantly, we are the first to demonstrate that conditional deletion of Wnt5a in mature osteoblasts and osteocytes by the Cre-loxP system could not significantly prevent cartilage degeneration of mice with ACLT-induced OA, as indicated by comparable histological scores of cKO and control littermates.

Generally, cartilage degeneration is considered both the main phenotype and central pathological process of OA (22). However, increasing evidence from recent studies revealed that cartilage deterioration can be preceded by abnormal remodeling of the subchondral bone (23, 24). The interaction of the bone-cartilage interface at multiple levels plays a crucial role in OA development (25), but the cellular and molecular events that occur between the cartilage and subchondral bone during this process are still unclear. Given the results of previous studies (10-13), especially overexpression of Wnt5a in both cartilage and subchondral bone in OA samples and the dual effects of Wnt5a in cartilage catabolism and subchondral bone remodeling, we proposed that Wnt5a has a crucial role in OA development. In this context, the main aim of the present study was to uncover the role of Wnt5a in mature osteoblasts during the degradation of cartilage in the context of OA development.

However, two unexpected results were found in the present study. First, the global histological score showed no significant difference between cKO mice and their control littermates, indicating that Wnt5a deficiency in osteoblasts cannot prevent cartilage degeneration in this model. Global histological changes were assessed by scoring systems evaluating articular cartilage structure, proteoglycan content, cellular appearance, tidemark integrity, and osteophyte formation, which are determined by various factors in addition to those related to subchondral bone osteoblasts. Thus, cell-specific expression changes of a single gene in bone may not sufficiently affect the biochemical or biomechanical environment of cartilage. In an animal study by Ziemian *et al*. (25), the loss of estrogen receptor α in bone resulted in an osteopenic subchondral bone phenotype but did not directly affect cartilage health. In this context, our findings in subchondral bone showed no significant difference between cKO and control littermates-another unexpected result of the present study. As shown in our micro-CT results, Wnt5a deficiency in osteoblasts does not affect the bone mass or microarchitecture of the tibia subchondral bone of either intact or ACLT knees. OA is associated with early bone loss, followed by densification of the subchondral plate and loss of cartilage. Subchondral densification is a late event in OA that involves only the subchondral plate and calcified cartilage; the subchondral cancellous bone beneath the subchondral plate may show osteopenia (3). In the present study, at 8 weeks after ACLT, BMD, Tb.N, and BV/TV values of the subchondral trabecular bone from OA joints were substantially lower than those from intact joints, while Tb.Sp and SMI values exhibited the opposite trend. With the exception of Tb.Sp in cKO mice, values showing an increased trend in ACLT knees compared with intact knees did not reach statistical significance. However, this mild change did not confirm that osteoblast-specific deficiency of Wnt5a affects the deterioration of subchondral bone in this OA model. 

Previous studies indicated a complicated role of Wnt5a in osteoblast and osteoclast differentiation and function. Wnt5a has been identified as a key regulator of osteoblast function and osteogenesis, (27-29) and a major constituent in osteoblastic differentiation stimulated by either physiological or other factors (30, 31). Moreover, calvarial osteoblast-like cells isolated from *Wnt5a*^-/-^ mice showed impaired mineralization even after treatment with bone morphogenic protein 2 (32). Osteoblast-lineage cells from Wnt5a-deficient mice exhibit reduced Wnt/β-catenin signaling, which impaired osteoblast differentiation and enhanced adipocyte differentiation (33). Notably, another study using the Cre-loxP system to knock out Wnt5a in osterix (Osx)-expressing cells reported impaired bone formation and decreased bone mass in trabecular bone (34). In the present study, the micro-CT results show no significant difference between cKO mice and control littermates, either in ACLT or intact joints. Furthermore, we assessed the pace of skeletal maturation of mice on postnatal day 1 by whole-mount skeletal staining (supplementary Figure 1), which indicated no obvious difference in skeletal development between cKO and control littermates. These somewhat contradictory results might be due to the differences in target cells that carried Cre recombinase. Briefly, Osx is mainly expressed by osteolineage-restricted progenitors (35-37) and continues to be expressed as these cells divide and differentiate into osteoblasts. Osteoblasts express Col1a1 at an immature stage, followed by OC when they are fully mature. Accordingly, Osx-Cre would affect a much wider range of osteoblast-lineage cells than OC and therefore showed greater impairment of osteogenesis than OC. However, Wnt5a and its receptor, receptor tyrosine kinase-like orphan receptor 2, mediate the effects of osteoblasts on osteoclastogenesis. Osteoblast-lineage cells express Wnt5a, whereas osteoclast precursors express Ror2. Mice deficient in either Wnt5a or Ror2 and those with either osteoclast precursor-specific Ror2 deficiency or osteoblast-lineage cell-specific Wnt5a deficiency showed impaired osteoclastogenesis (32). Similarly, Wnt5a/Ror2 signaling increased *Cxcl12* and *Rankl* gene expression induced by JNK and Ca^2+^/NFAT signaling pathways, leading to activation of osteoclast differentiation and enhanced subchondral bone turnover (14). Therefore, we propose that during OA development in this model, bone loss in the subchondral bone was due to activated bone remodeling, whereas in cKO mice, both the osteogenic activity of osteoblasts and osteoclast differentiation which greatly promote elevated bone re-modeling were inhibited. These events ultimately lead to similar levels of these parameters in cKO mice and their littermates.

One limitation of the present study is that the Cre-loxP system used is not time-specific, as the mice carrying Cre and floxed *Wnt5a* would show osteoblast-specific knockout of *Wnt5a* before birth. Although no significant differences were found in intact knees between cKO mice and control littermates in any of the parameters observed, this factor may have influenced the results of this study. An advanced knockout strategy, such as the ERT-2-cre system, could be used for further elucidation of the specific and precise role of osteoblast-produced Wnt5a in OA development.

## Conclusion

Based on the findings in the present study, Wnt5a deficiency in mature osteoblasts is insufficient to block OA degeneration in surgically induced mice. These results demonstrate the advantage of cell-specific genetically modified mice, which can provide a more concise story of events during the pathological process of the modelled disease. In this context, the negative result in the present study is also a valuable reference for future research focused on the cell-specific role of Wnt5a in certain physiological or pathological processes. More studies, such as alterations in cell lineages carrying Cre recombinase or specific deletion of target genes in chondrocytes, are needed.

## Authors’ Contributions

LJ F, L Z, and FM T designed the study. LJ F, XH F, Y L, LT S, and YY Z conducted the study and collated the data. YP H and XL H performed statistical analysis. LJ F drafted the manuscript. LZ and FM T revised the manuscript. All authors approved the final version.

## Statement of Ethics

The animal study protocol was approved by the Institutional Animal Care and Use Committee of North China University of Science and Technology (LX2019034), Tangshan, China. 

## Conflicts of Interest

The authors declare that they have no conflicts of interest. 
